# Plasmodium berghei PIMMS2 Promotes Ookinete Invasion of the Anopheles gambiae Mosquito Midgut

**DOI:** 10.1128/IAI.00139-17

**Published:** 2017-07-19

**Authors:** Chiamaka V. Ukegbu, Karolina A. Akinosoglou, George K. Christophides, Dina Vlachou

**Affiliations:** Laboratory of Insect Immunogenomics, Department of Life Sciences, Imperial College London, London, United Kingdom; University of South Florida

**Keywords:** malaria, mosquito midgut invasion, Plasmodium, host-parasite interactions, subtilisins

## Abstract

Mosquito midgut stages of the malaria parasite present an attractive biological system to study host-parasite interactions and develop interventions to block disease transmission. Mosquito infection ensues upon oocyst development that follows ookinete invasion and traversal of the mosquito midgut epithelium. Here, we report the characterization of PIMMS2 (Plasmodium invasion of mosquito midgut screen candidate 2), a Plasmodium berghei protein with structural similarities to subtilisin-like proteins. *PIMMS2* orthologs are present in the genomes of all plasmodia and are mapped between the subtilisin-encoding genes *SUB1* and *SUB3*. P. berghei PIMMS2 is specifically expressed in zygotes and ookinetes and is localized on the ookinete surface. Loss of PIMMS2 function through gene disruption by homologous recombination leads to normal development of motile ookinetes that exhibit a severely impaired capacity to traverse the mosquito midgut and transform to oocysts. Genetic complementation of the disrupted locus with a mutated *PIMMS2* allele reveals that amino acid residues corresponding to the putative subtilisin-like catalytic triad are important but not essential for protein function. Our data demonstrate that PIMMS2 is a novel ookinete-specific protein that promotes parasite traversal of the mosquito midgut epithelium and establishment of mosquito infection.

## INTRODUCTION

Malaria remains a devastating disease despite continuous efforts for control and prevention. The Plasmodium dual life cycle in the vertebrate and mosquito hosts requires invasion or traversal of various types of host cells by specialized parasite invasive forms (1). Soon after ingestion of a gametocyte-containing blood meal by a female Anopheles mosquito, male and female gametes are formed in the mosquito midgut lumen. Gametes then fuse to produce a zygote which differentiates into a motile ookinete. To establish a mosquito infection an ookinete must successively traverse two physical barriers, the chitinaceous peritrophic matrix that surrounds the blood bolus and the midgut epithelium. At the basal subepithelial space, the ookinete differentiates into a replicative oocyst where thousands of sporozoites are produced. Sporozoites are released into the mosquito hemocoel and invade the salivary gland (1, 2). Inoculation of sporozoites residing in the salivary gland lumen into a vertebrate host occurs during a mosquito bite.

The gametocyte-to-oocyst transition is completed within approximately 24 h after mosquito ingestion of the infected blood. During this stage, significant parasite losses occur that result in only a small number of ookinetes succeeding to transform to oocysts and establishing a mosquito infection (3). Indeed, in most cases, transmission is terminated at this stage, which therefore represents an ideal target for the development of transmission-blocking interventions (3).

Ookinete midgut traversal and transformation to oocysts is associated with *de novo* protein synthesis in developing ookinetes, which are thought to be important for host cell recognition, binding, and motility. They include the circumsporozoite and TRAP-related protein (CTRP [4, 5]), chitinase (CHT1 [6]), the secreted ookinete adhesive protein (SOAP [7]), the von Willebrand factor A domain-related protein (WARP [8]), and the perforin-like proteins 3 (PPLP3) (9) and PPLP5 (10). Our developmental transcriptome analysis of the murine malaria parasite P. berghei in the midgut of Anopheles gambiae mosquitoes has previously highlighted a number of additional ookinete-expressed genes encoding proteins putatively involved in ookinete development and midgut traversal (11). Here, we report the characterization of one of these proteins, PIMMS2, which is specifically expressed in the zygote and ookinete. PIMMS2 shows structural similarity to subtilisin-like proteins and localizes on the ookinete surface. We use homologous recombination to disrupt the *PIMMS2* genomic locus and study the function of the protein during parasite development and mosquito infection, and we reveal that PIMMS2 promotes midgut epithelium traversal. We also use genetic complementation to investigate the relevance of the subtilisin-like structural homology to the function of PIMMS2 and show that conserved amino acid residues corresponding to the catalytic triad of other known subtilisin-like proteins are important but not essential for the function of PIMMS2.

## RESULTS

### Identification of P. berghei PIMMS2 (PbPIMMS2).

**FIG 1:**
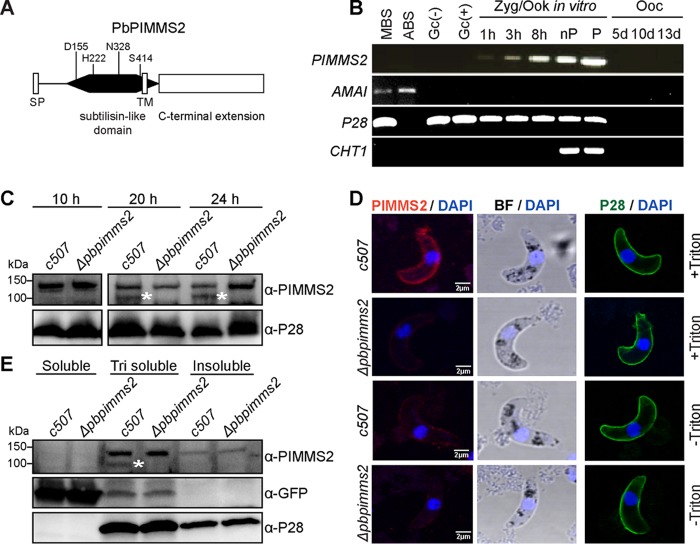
Domain architecture, expression, and localization of PbPIMMS2. (A) PIMMS2 domain architecture. The predicted signal peptide (SP), subtilisin-like domain, transmembrane domain (TM), and C-terminal extension are shown. The amino acid residues D155, H222, and S414, which correspond to the catalytic triad of subtilases as well as the conserved N328 amino acid residue, are also shown. (B) Expression profile of Pb*PIMMS2* revealed by RT-PCR analysis in asexual blood stages (ABS) of the non-gametocyte-producing strain HPE, mixed-blood stages (MBS), activated (+) and nonactivated (−) gametocytes (Gc), 1-h, 3-h, and 8-h zygotes (Zyg), nonpurified (nP) and purified (P) *in vitro*-produced ookinetes (Ook), and *in vivo*-developed 5-, 10-, and 13-day (d) oocysts (Ooc) in A. gambiae mosquitoes. *P28*, *AMA1*, and *CHT1* served as stage-specific and loading controls. (C) Western blot analysis of total *c507* and Δ*pbpimms2* ookinete extracts collected at 10, 20, and 24 h after gametocyte activation using the α-PIMMS2 antibody. Antibody against P28 was used as an internal control. Proteins were separated on a 4 to 20% SDS-PAGE gel. The approximately 100-kDa PIMMS2-specific bands are marked with asterisks. (D) Immunofluorescence assays of Triton-permeabilized (+ Triton) and nonpermeabilized (− Triton) *c507* and Δ*pbpimms2* ookinetes stained with 4′,6-diamidino-2-phenylindole (DAPI) (blue) and α-PIMMS2 (red). Bright-field (BF) visualization is also shown. In the third column of images, staining with α-P28 (green) antibody of the same ookinete preparations was used as a control. All images were taken from confocal sections of fixed parasites. The scale bar is set at 2 μm. (E) Western blot analysis of fractionated total protein extracts of *c507* and Δ*pbpimms2* ookinetes using the α-PIMMS2 antibody. Antibodies against GFP and P28 were used as controls. Proteins were separated on a 4 to 20% SDS-PAGE gel. The 100-kDa PIMMS2-specific band is marked with an asterisk.

A transcriptomic analysis of P. berghei in the A. gambiae midgut previously identified several genes expressed during ookinete development and midgut epithelium traversal (11). One of these genes, *PBANKA_1106900*, is mapped between genes encoding two known subtilisin-like proteases, *SUB1* and *SUB3*, and encodes an 836-amino-acid-long protein. We named this gene *PIMMS2*, for midgut invasion ookinete protein screen candidate 2. Bioinformatics analysis of the deduced protein sequence, using the open-access tools Phyre^2^ (12), Phobius, and PolyPhobius (13), predicted with high probability two hydrophobic regions of alpha-helix structure encompassing the amino acid residues 6 to 25 and 416 to 427, respectively (Fig. 1A). Additionally, TargetP (14) predicted with high reliability a cleavage site at amino acid residue 30, altogether suggesting an N-terminal signal peptide and a transmembrane domain located at the center of the protein.

Protein sequence analysis predicted a peptidase S8 domain (IPR000209) that ends shortly after the predicted transmembrane domain and is followed by a long C-terminal extension (CTE) with no known domain homology (Fig. 1A). Indeed, three-dimensional homology modeling using Phyre^2^ revealed a highly significant (100% confidence) structural similarity of the predicted peptidase S8 domain region to Plasmodium vivax SUB1 (PvSUB1) (15) and Plasmodium falciparum SUB1 (16). The homology model, built upon the highest-scoring template (PDB code 4TR2) corresponding to the catalytic domain of PvSUB1, comprises 9 α-helices and 10 β-strands arranged in the α/β-fold–αβ-sheet sandwiched between two prominent surface α-helices, which is typical for subtilisin-like proteins (see Fig. S1 in the supplemental material).

PIMMS2 sequence alignments with PvSUB1 and the bacterial subtilisin BPN′ (17) identified several conserved amino acid residues within the predicted subtilisin-like domain, including D155, H122, and S414, which correspond to the catalytic triad of subtilisin-like serine proteases (Fig. S1 and S2). A conserved asparagine residue, N328, which serves in stabilizing the putative oxyanion hole, was also identified. However, the conserved motifs HGT and GTS, which encompass the catalytic His and Ser residues of subtilases, respectively, are not present in PIMMS2, suggesting that the putative catalytic triad is nonfunctional; thus, PIMMS2 is a potential pseudoenzyme.

Full-length protein comparisons revealed high sequence identity between PbPIMMS2 and predicted proteins in two other murine parasites, Plasmodium yoelii (81%) and Plasmodium chabaudi (69%), designated PyPIMMS2 (PYYM_1109100) and PcPIMMS2 (PCHAS_1106600), respectively (Fig. S3). Orthologs of PIMMS2 were also identified in human parasites with known genome sequences, including P. falciparum (PfPIMMS2; PF3D7_0507300), P. vivax (PvPIMMS2; PVX_097925), and P. knowlesi (PkPIMMS2; PKNH_1026300); however, the overall identity, although remaining higher in the subtilisin-like domain regions, drops substantially to 18 to 20%. A CTE is detected in all PIMMS2 orthologs. In all six Plasmodium species examined, the *PIMMS2* gene is mapped in a highly syntenic genomic locus between *SUB1* and *SUB3*.

The subtilisin-like domain architecture of PbPIMMS2 is conserved only in PvPIMMS2; the rest of the PIMMS2 orthologs appear to lack one or more of the conserved amino acid residues or motifs (Fig. S3). Of all the PIMMS2 orthologs, PfPIMMS2 is the most divergent, lacking the aspartic acid residue that is part of the putative catalytic triad despite high conservation of surrounding amino acid residues. The predicted catalytic histidine residue is also absent from PfPIMMS2 as well as from PyPIMMS2 and PcPIMMS2. The HGT motif at the predicted catalytic histidine site is present only in PvPIMMS2 and PkPIMMS2, while all of the orthologs lack the GTS motif at the site of the catalytic serine, altogether reinforcing the view that PIMMS2 is a subtilisin-like pseudoenzyme. The CTE is highly divergent, especially between murine and human malaria parasites.

### Expression profiling of *PIMMS2*.

We examined the temporal expression pattern of P. berghei *PIMMS2 in vivo* and in *in vitro*-cultured parasite stage-specific populations using reverse transcription-PCR (RT-PCR) (Fig. 1B). The results revealed that *PIMMS2* transcripts are limited to mosquito midgut stages. Transcripts were as early as 1 h after gametocyte activation and peaked as the ookinete development progressed. No *PIMMS2* transcripts were detected in the oocyst.

We raised a polyclonal antibody against the subtilisin-like domain of PIMMS2 extending from Ile117 to Gly475 and used it in immunofluorescence assays and Western blotting. Ookinetes that lacked *PIMMS2* (Δ*pbpimms2*), generated as described in the next section, were used as a control. In Western blots of total protein extracts prepared from *in vitro*-produced and -purified ookinetes, despite background signal, the anti-PIMMS2 antibody detected a specific band of approximately 100 kDa, corresponding to a mature full-length protein that was absent from Δ*pbpimms2* ookinetes (Fig. 1C). This band was detected as early as 10 h after gametocyte activation and peaked in mature ookinetes at 20 and 24 h after gametocyte activation, corroborating the progressive accumulation of *PIMMS2* transcripts revealed by RT-PCR.

Immunofluorescence assays revealed a prominent surface distribution of PIMMS2 in *in vitro*-produced ookinetes that were permeabilized using Triton X-100 (Fig. 1D). This signal was largely abolished in nonpermeabilized ookinetes, suggesting that the subtilisin-like domain of PIMMS2, against which the antibody was raised, is located on the internal side of the ookinete surface. The surface distribution of P28, which was used as a control, showed no difference between permeabilized and nonpermeabilized ookinetes, and it was not affected by the disruption of PIMMS2.

To further investigate the putative membrane association of PIMMS2, purified ookinetes were fractionated based on protein solubility, and the fractions were analyzed by Western blotting (Fig. 1E). The 100-kDa PIMMS2-specific band was detected only in the Triton-soluble fraction, consistent with the surface localization of PIMMS2 in immunofluorescence assays and the prediction of a transmembrane domain. Supporting the quality of the fractionated samples, green fluorescent protein (GFP) that is constitutively expressed in the cytoplasm of the *ANKA 507m6cl1* reference line (18), here termed the *c507* line, was mostly found in the cytoplasmic fraction, while P28 was detected in both the Triton-soluble and -insoluble fractions.

### PIMMS2 loss-of-function analysis.

**FIG 2:**
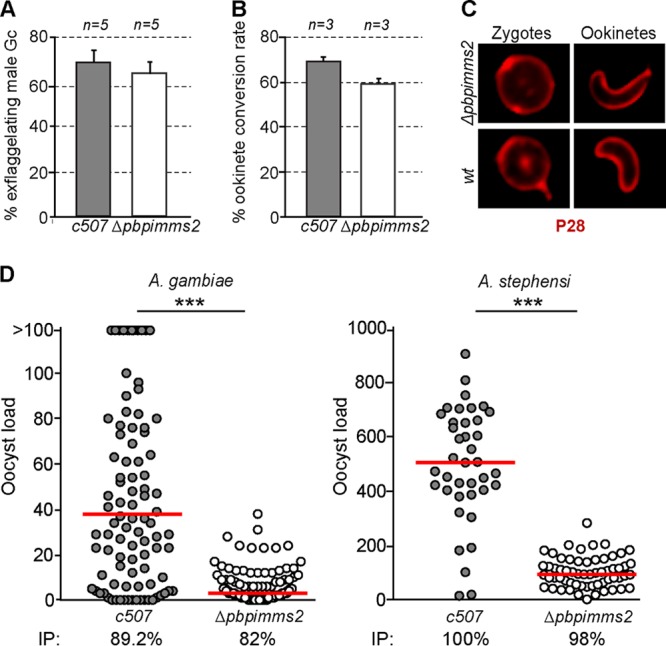
Phenotypic analysis of Δ*pbpimms2* mutant parasites. (A) Exflagellation assays showing the percentage of *c507* and Δ*pbpimms2* male gametocytes forming exflagellation centers. Five independent biological replicates were performed (*n* = 5). (B) Macrogamete-to-ookinete conversion rates of *c507* and Δ*pbpimms2* parasite lines determined as the percentage of ookinetes to macrogametes plus ookinetes. Three independent biological replicates were performed (*n* = 3). (C) P28 distribution in zygotes and ookinetes of *c507* and Δ*pbpimms2* parasite lines. (D) Oocyst loads of *c507* and Δ*pbpimms2* parasites in A. gambiae (left) and A. stephensi (right) mosquitoes. The mosquito infection prevalence (IP) is shown below each graph. The median oocyst load is shown with a red line. Stars indicate statistical significance determined with the Mann-Whitney U test for the oocyst loads and chi-squared goodness-of-fit test for the infection prevalence (***, *P* < 0.0001).

We generated P. berghei mutants lacking a functional *PIMMS2* gene by replacing a large part of the *PIMMS2* coding region with a modified Toxoplasma gondii pyrimethamine resistance cassette in the *c507* GFP-expressing parasite reference line (18), as shown in Fig. S4A and described in Materials and Methods. Two clonal lines were generated from respective independent transfections. Integration of the disruption cassette and disruption of the *PIMMS2* gene were verified by PCR of genomic DNA (Fig. S4B) and Southern analysis of pulsed-field separated chromosomes (Fig. S4C). Phenotypic analysis revealed that activation of male gametogenesis, measured by the formation of exflagellation centers, was normal in Δ*pbpimms2* parasites (Fig. 2A). The ookinete conversion rate of Δ*pbpimms2* parasites was also comparable to that of *c507* controls (Fig. 2B), and both macrogametes and ookinetes displayed normal morphology and surface distribution of P28 (Fig. 2C).

We assessed the ability of Δ*pbpimms2* parasites to develop to oocysts in both A. gambiae and A. stephensi mosquitoes fed on mice infected with Δ*pbpimms2* or control *c507* parasites. The numbers of oocysts were determined 10 days postinfection (dpi). The results showed that the oocyst numbers were significantly reduced in Δ*pbpimms2* compared to *c507* controls, reflecting a strong defect in either ookinete motility or midgut invasion capacity (Fig. 2D and Table S1). Despite the strong reduction in oocyst numbers, the infection prevalence was not significantly different between Δ*pbpimms2* and *c507* controls. The numbers of both oocyst and salivary gland Δ*pbpimms2* sporozoites were also significantly reduced compared to those of *c507* controls, presumably reflecting the reduced oocyst numbers (Table S2). The Δ*pbpimms2* sporozoites were nevertheless able to infect susceptible BL6/C56 recipient mice as revealed by bite-back experiments using either A. gambiae or A. stephensi mosquitoes at days 18 and 21 postinfection (Table S2).

### PIMMS2 functions during midgut traversal.

**FIG 3:**
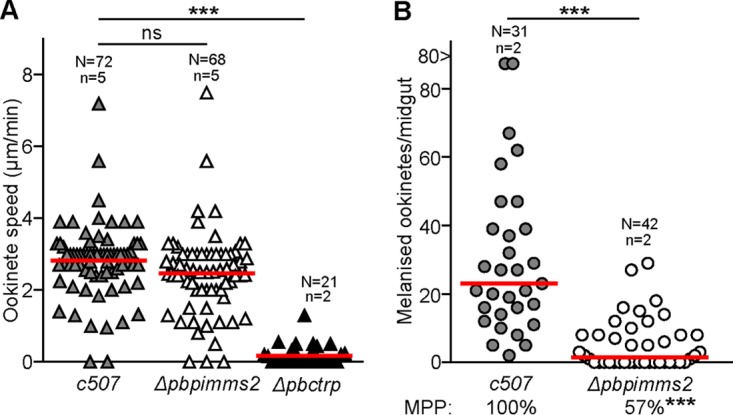
Motility and invasion assays of Δ*pbpimms2* ookinetes. (A) Ookinete motility assays of Δ*pbpimms2*, *c507*, and Δ*pbctrp* parasites. Ookinetes were allowed to translocate on Matrigel prepared slides, and their speed was calculated. *P* values were determined by two-tail Student *t* test. Horizontal red lines indicate the mean ookinete speed in micrometers per minute. N is the number of ookinetes, and n is the number of biological replicates. (B) Δ*pbpimms2* ookinete invasion assay in L3-5 A. gambiae mosquitoes. Distribution and median (red lines) of melanized ookinete load intensities are shown. N is the number of midguts tested; n is the number of independent biological replicates. The melanized parasite prevalence (MPP) is shown below the graph. Stars indicate statistical significance determined with the Mann-Whitney U test for the melanized parasite numbers and chi-squared goodness-of-fit test for the melanized parasite prevalence (***, *P* < 0.0001; ns, not significant).

We carried out motility assays to examine whether the Δ*pbpimms2* phenotype is due to defective ookinete motility. Time-lapse microscopy revealed that Δ*pbpimms2* mutant ookinetes do not exhibit impaired motile behavior or capacity to translocate *in vitro* compared to control *c507* ookinetes (Fig. 3A). We next examined whether the Δ*pbpimms2* ookinetes fail to traverse the mosquito midgut epithelium or are eliminated after they traverse the epithelium prior to their development to oocysts. Infections with Δ*pbpimms2* and *c507* control parasites were carried out in A. gambiae L3-5 mosquitoes that are known to melanize P. berghei ookinetes immediately after they traverse the epithelium upon exiting into the basal subepithelial space. Therefore, this assay provides a powerful means of quantifying the ookinete invasive ability. To control for variation in gametocyte numbers, membrane feeds of equal numbers of *in vitro*-cultured ookinetes were performed. The results revealed that there were significantly lower numbers of melanized Δ*pbpimms2* ookinetes compared to *c507* controls, indicating that the Δ*pbpimms2* phenotype is due to the failure of mutant ookinetes to traverse the midgut epithelium (Fig. 3B and Table S1). A strong reduction in the prevalence of melanized parasites was also observed. Taken together, our data suggest that PIMMS2 plays a role in ookinete traversal of the mosquito midgut.

**FIG 4:**
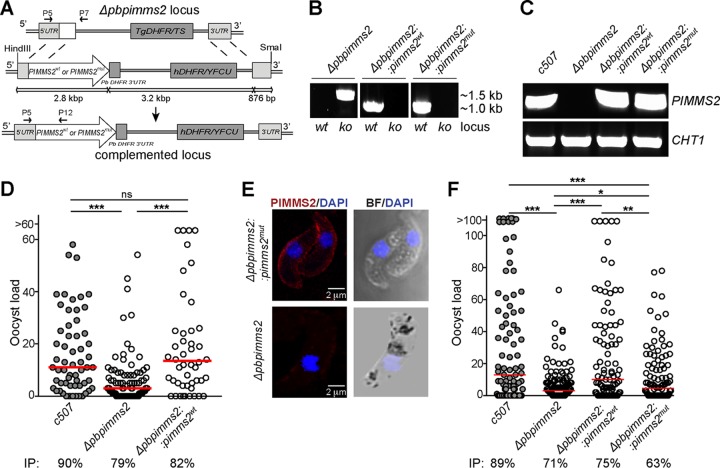
Genetic complementation of Pb*PIMMS2*. (A) Schematic representation of the generation of the genetic complementation parasite. In Δ*pbpimms2* parasites, the *PIMMS2* locus is restored by homologous recombination with either the wt allele (*PIMMS2*^*wt*^) or a modified allele (*PIMMS2*^*mut*^) carrying alanine mutations in the predicted catalytic triad residues. (B) Genotyping of the Δ*pbpimms2*::*pimms2*^*wt*^ and Δ*pbpimms2*::*pimms2*^*mut*^ transgenic parasites by PCR on genomic DNA, showing restoration of the *PIMMS2* gene in the complemented Δ*pbpimms2* locus with the *PIMMS2*^*wt*^ and *PIMMS2*^*mut*^ alleles, respectively. The panels represent photos taken from different areas of the same gel that were then joined together. (C) RT-PCR analysis showing *PIMMS2*^*wt*^ and *PIMMS2*^*mut*^ transcripts in Δ*pbpimms2*::*pimms2*^*wt*^ and Δ*pbpimms2*::*pimms2*^*mut*^ ookinetes, respectively. Detection of *PIMMS2* transcripts in *c507* but not in Δ*pbpimms2* ookinetes was used as negative and positive controls, respectively. (D) Oocyst load and distribution in the midguts of A. gambiae mosquitoes infected with Δ*pbpimms2*::*pimms2*^*wt*^ transgenic parasites at day 10 pi. Infections with *c507* and Δ*pbpimms2* parasites served as controls. The median oocyst load is shown with a red line. The mosquito infection prevalence (IP) is shown below the graph. (E) Immunofluorescence assays of Triton-permeabilized Δ*pbpimms2*::*pimms2*^*mut*^ ookinetes stained with DAPI (blue) and α-PIMMS2 (red). Bright-field (BF) visualization is also shown. Immunofluorescence assay of Δ*pbpimms2* ookinetes was used as a negative control. All images were taken from confocal sections of fixed parasites. The scale bar is set at 2 μm. (F) Oocyst load and distribution in the midguts of A. gambiae mosquitoes infected with Δ*pbpimms2*::*pimms2*^*mut*^ parasites at day 10 pi. Infections with *c507*, Δ*pbpimms2*, and Δ*pbpimms2*::*pimms2*^*mut*^ parasites served as controls. The mosquito infection prevalence is shown below the graph. The median oocyst load is shown with a red line. Asterisks indicate statistical significance determined with the Mann-Whitney U test for the oocyst loads and chi-squared goodness-of-fit test for the infection prevalence (***, *P* < 0.001; **, *P* < 0.01; *, *P* < 0.05; ns, nonsignificant).

To validate the revealed function of PIMMS2, we reintroduced a *PIMMS2* wild-type (wt) allele in the Δ*pbpimms2* mutant parasites and examined whether the oocyst numbers can be restored (Fig. 4). Reconstitution of the *PIMMS2* locus was verified by PCR on genomic DNA of clonal parasite lines (Fig. 4A and B), while *PIMMS2* transcripts were detected in Δ*pbpimms2*::*pimms2*^*wt*^ transgenic ookinetes by RT-PCR analysis (Fig. 4C). The capacity of Δ*pbpimms2*::*pimms2*^*wt*^ parasites to traverse the A. gambiae mosquito midgut and develop to oocysts was assessed 10 dpi. As infection controls, *c507* and Δ*pbpimms2* parasites were used. The results showed that the Δ*pbpimms2*::*pimms2*^*wt*^ oocyst numbers were comparable to the numbers of *c507* and significantly higher than the numbers of Δ*pbpimms2* parasites (Fig. 4D and Table S3).

### Mutation of PIMMS2 putative catalytic triad.

The absence of typical subtilisin amino acid residues/motifs in PbPIMMS2 in conjunction with extensive polymorphisms in such amino acid residues/motifs in other Plasmodium PIMMS2 orthologs suggested that PbPIMMS2 is catalytically inactive. To further investigate this, we performed genetic complementation experiments whereby the Δ*pbpimms2* locus was reconstituted with a mutated *pimms2*^*mut*^ allele in which the conserved Asp155, His222, and Ser414 residues, which make up the catalytic triad of subtilases, were replaced with alanine residues by site-directed mutagenesis. Mutation of the catalytic triad previously has been shown to lead to loss of the subtilase enzymatic function (19, 20). The design was similar to that presented in Fig. 4A for Δ*pbpimms2* complementation with a *PIMMS2*^*wt*^ allele. Reconstitution of the *PIMMS2* locus was verified by PCR on genomic DNA of a clonal Δ*pbpimms2*::*pimms2*^*mut*^ parasite line (Fig. 4B). *PIMMS2*^*mut*^ transcripts were detected in Δ*pbpimms2*::*pimms2*^*mut*^ ookinetes by RT-PCR analysis (Fig. 4C), and immunofluorescence analysis confirmed that the PIMMS2^mut^ protein exhibits a distribution pattern similar to that of the wt PIMMS2 protein (Fig. 4E).

Phenotypic analysis showed that the ookinete conversion rate of Δ*pbpimms2*::*pimms2*^*mut*^ parasites was comparable to that of *c507* parasites (data not shown). We assessed the ability of Δ*pbpimms2*::*pimms2*^*mut*^ parasites to traverse the midgut of A. gambiae mosquitoes and develop to oocysts quantified 10 dpi. Mosquito infections with *c507*, Δ*pbpimms2*, and Δ*pbpimms2:*:*pimms2*^*wt*^ parasites served as a control. The results showed that the Δ*pbpimms2*::*pimms2*^*mut*^ parasites exhibited oocysts numbers that were significantly lower than those of the *c507* and Δ*pbpimms2*:*pimms2*^*wt*^ parasites but higher than those of the Δ*pbpimms2* parasites (Fig. 4F and Table S3). These data suggest that the amino acid residues that form the putative catalytic triad of subtilases are important but not essential for the function of PIMMS2.

## DISCUSSION

Within about 24 h inside the mosquito, Plasmodium parasites must invade and traverse the midgut cell wall in order to successfully establish an infection, marked by the development of oocysts on the basal side of the midgut epithelium. The mechanisms underpinning invasion and traversal of the midgut epithelium by Plasmodium ookinetes have not been fully elucidated. Here, we show that P. berghei PIMMS2, an ookinete-specific protein exhibiting structural similarities to subtilisin-like proteins, is involved in mosquito midgut traversal. Previously characterized subtilisin-like proteins in Plasmodium include SUB1, SUB2, and SUB3, which are exclusively or primarily expressed during asexual development (21). SUB1 and SUB2 are shown to play important roles in merozoite invasion of erythrocytes through proteolytic processing of merozoite proteins, such as MSP1 and AMA1, as well as in merozoite egress from erythrocytes through proteolytic cleavage of the SERA proteins mediated by SUB1 (22–27). P. berghei SUB2 is also found in ookinetes, where it has been suggested to play a role in modifying the cytoskeletal network of the invaded mosquito midgut cells (28). SUB3 is the least characterized member of the family, and it is thought to function in host immune evasion (29, 30).

Despite the structural similarity of PbPIMMS2 to the P. vivax SUB1 and the partial loss of function upon mutation of the conserved amino acid residues that make up the subtilase catalytic triad, several sequence discrepancies between PIMMS2 and previously characterized subtilases do not allow any inference for a PIMMS2 enzymatic function. In addition, most PIMMS2 orthologs in other plasmodia lack one or more of the residues involved in the putative catalytic triad. Additional biochemical and structural data are needed to elucidate the function of PIMMS2 during mosquito midgut traversal. It has been suggested that enzymes that have lost residues important for catalytic activity take up regulatory roles (31, 32). Therefore, in the same manner that the papain protease domain of SERA5, despite losing its protease activity, is important for parasite development (32), the PIMMS2 subtilisin domain may not be catalytically active but is important for its function. Indeed, it is possible that PIMMS2 has assumed a nonenzymatic regulatory function, including modulation of the activity of homologous enzymes, e.g., through competition for substrate binding or substrate protection from cleavage and signaling upon interaction with the substrate or other proteins. It is worth noting that SUB2, which is also suggested to play a role during mosquito midgut traversal (28), is coexpressed with PIMMS2 in P. berghei ookinetes. Therefore, it is possible that a catalytically nonactive PIMMS2 regulates the activity of SUB2 during this process.

Although the precise function of PbPIMMS2 in midgut traversal remains unknown, it is tempting to speculate that it is linked to the actomyosin motor that drives parasite invasion as well as motility and is located in the pellicular space between the plasma membrane and the inner membrane complex (33). PIMMS2 may play a role in the cytoskeletal dynamics by interacting with proteins involved in the invasion machinery to eventually promote invasion. Ookinete invasion of the midgut epithelium is shown to occur largely via an intracellular route. After penetrating the apical epithelial cell membrane, ookinetes cross the cell or, indeed, many cells successively before exiting into the subepithelial space (28, 34). The traversed cells undergo apoptosis and are expelled from the epithelial cell lining. However, it has been shown that ookinetes lacking the major surface proteins P25 and P28 can achieve epithelium traversal using an intercellular route that does not elicit cell apoptosis and expulsion, thus exhibiting a partial infection phenotype similar to that of *PIMMS2* mutants (35, 36). Therefore, it is pertinent to speculate that PIMMS2 is involved in epithelial cell invasion and that the few *PIMMS2* mutant parasites establishing midgut infection achieve this by following an intercellular route for epithelium traversal that does not require the function of PIMMS2. Similar strong yet incomplete midgut infection phenotypes are displayed by P. berghei ookinetes lacking additional surface-associated or secreted proteins, including SOAP (7), cell traversal protein for ookinetes and sporozoites (CelTOS) (37), and PSOP2 and PSOP9 (38). It is possible that these partial phenotypes are indeed associated with alternative pathways for midgut traversal (39).

In invasive parasite stages, invasion has been tightly linked to gliding motility (40), and ookinetes that display defective motility also are shown to be invasion defective, as observed in the CTRP and CDPK3 null mutants (4, 41). If PIMMS2 interacts with components of the actomyosin machinery, our data suggest that these interactions do not affect the ookinete motility, and that indeed invasion and motility can be independent. This has been shown previously in sporozoites, another invasive Plasmodium stage, whereby HSP20 functions in sporozoite migration but not in hepatocyte invasion (42).

In contrast to the majority of subtilases belonging to the subtilisin family (17), including Plasmodium SUB1 and SUB3 (43), PIMMS2 possesses a C-terminal extension (CTE) downstream of the predicted subtilisin-like domain. PfSUB2 also has a CTE following its transmembrane domain (44). However, the CTE of PIMMS2 is uncommonly long, encompassing approximately half of the mature protein. Such extensions are indeed rare in the subtilisin family of subtilases, with most of them found in the eukaryotic kexin family of subtilases, such as prohormone convertases (45) and mammalian furin (46). CTEs have been proposed to function in enzyme stability, localization, and trafficking, as well as in substrate binding and recognition (47–49). The function of the CTE in PbPIMMS2 is another interesting avenue for further investigation that could provide additional data to understand the function of PIMMS2.

The discovery of PIMMS2 can assist in the effort to dissect the molecular processes driving ookinete traversal of the mosquito midgut epithelium and delineate similarities and differences of this with merozoite invasion and/or egress of host erythrocytes and hepatocytes. Additionally, mosquito midgut traversal by ookinetes is a highly limiting step in the malaria transmission cycle, and understanding the function of proteins involved in this process could inform the development of interventions aiming to block disease transmission (50). A number of ookinete surface proteins, some of which are involved in this process, have been proposed and/or indeed studied as targets for transmission-blocking vaccines. The identification of PIMMS2 adds another candidate to this list.

## MATERIALS AND METHODS

### Ethics statement.

All animal procedures were carried out in accordance with the Animal (Scientifics Procedures) Act 1986 under the UK Home Office licenses PPL70/7185 and PPL70/8788. The numbers of animals used were minimized by incorporation of the most economical protocols. Opportunities for reduction, refinement, and replacement of animal experiments were constantly considered and implemented where appropriate.

### Parasite cultivation and mosquito infections.

The P. berghei strains ANKA 2.34, non-gametocyte producer HPE, and GFP-expressing reference line ANKA 507m6cl1 (18) were propagated in CD1 mice. Parasite cultivation and purification was carried out as previously described (51). A. gambiae and A. stephensi mosquitoes were infected with P. berghei by direct blood feeding on anesthetized infected mice or through ookinete membrane feeding as previously described (52). For direct feeding, mice with parasitemia of 6 to 7% and gametocytemia of 1 to 2% were used.

### Transcriptional profiling using RT-PCR.

Total RNA was extracted from asexual blood stages (ABS) (HPE line), mixed blood stages (MBS), purified gametocytes, and *in vitro*-cultivated *c507* ookinetes using TRIzol reagent (Invitrogen). Gene-specific primers were designed using Primer 3 (v.0.4.0) and are presented in Table S4 in the supplemental material.

### Recombinant protein expression and purification and antibody production.

*PbPIMMS2*^*opt*^ comprised the complete P. berghei *PIMMS2* open reading frame (ORF), engineered by Life Technologies to contain codons allowing for optimized expression in Escherichia coli. The subtilisin-like domain (Ile117 to Gly475) was amplified by PCR from *PbPIMMS2*^*opt*^ using the primers P16 and P17 (Table S4). The PCR fragment was cloned by infusion cloning into a NotI-digested protein expression vector plasmid, pET-32b, which carries N- and C-terminal hexahistidine and N-terminal thioredoxin tags (Novagen) to generate pET-32b:*PbPIMMS2*^*opt*^CD.

Shuffle T7 E. coli cells (NEB) containing pET-32b:*PbPIMMS2*^opt^CD were grown at 30°C and induced with isopropyl-1-thio-β-d-galactopyranoside at 19°C for 16 h. Cells were harvested by centrifugation and lysed using BugBuster plus Lysonase (Novagen) supplemented with a cocktail of protease inhibitors (cOmplete EDTA-free; Roche). Cell debris was removed by centrifugation. The resulting histidine fusion protein in the soluble fraction was purified by cobalt affinity chromatography using TALON resins (Clontech) under native conditions in phosphate-buffered saline (PBS), pH 7.4. Bound proteins were eluted using 250 mM imidazole in PBS, pH 7.4. Protein samples were analyzed by SDS-PAGE to determine purity prior to its use in immunization.

A rabbit polyclonal antibody against the subtilisin-like domain of PbPIMMS2 (amino acids 117 to 475) was raised and purified from the serum of an immunized rabbit (Eurogentec).

### Western blot analysis and immunofluorescence assays.

Whole-cell lysates were extracted from purified ookinetes by boiling in 1× Laemmli buffer at 95°C for 10 min under reducing conditions. For fractionation, purified ookinetes were also subjected to hypotonic lysis in cold lysis buffer with 5 mM Tris-HCl, pH 7.5, supplemented with protease inhibitor to obtain the soluble fraction. Cells were spun down and the supernatant (soluble fraction) was removed. The pellet was washed twice in hypotonic lysis buffer, and the pellet resuspended in 50 mM Tris-HCl, pH 7.5, 150 mM NaCl, 1% Triton X-100 supplemented with protease inhibitor and incubated on ice for 20 min to obtain the Triton-soluble fraction. Cells were spun and the supernatant (Triton-soluble fraction) was removed. The pellet was washed twice with 50 mM Tris-HCl, pH 7.5, 150 mM NaCl, resuspended in 1× Laemmli buffer, and boiled at 95°C for 10 min to obtain the insoluble fraction. Protein samples of purified parasites were boiled under reducing conditions in 1× Laemmli buffer, and proteins were separated using 10% SDS-PAGE. Detection was performed according to standard procedures using α-PIMMS2, goat α-GFP (Rockland Chemicals), and mouse α-P28 monoclonal (13.1) antibodies at 1:100, 1:100, and 1:1,000 dilutions, respectively. Secondary horseradish peroxidase (HRP)-conjugated goat anti-rabbit IgG, goat anti-mouse IgG (Promega), and donkey anti-goat (Abcam) were used at 1:5,000, 1:10,000, and 1:5,000 dilutions, respectively.

For immunofluorescence assays (IFAs), 24-h mature ookinetes were fixed in 4% paraformaldehyde (PFA), smeared on a glass slide, and allowed to air dry. Fixed ookinetes were left nonpermeabilized or were permeabilized in 0.2% Triton X-100 prior to blocking in blocking buffer containing 1% bovine serum albumin (BSA), 1% goat serum, and antibody. α-PIMMS2 and α-P28 antibodies were used at dilutions of 1:100 and 1:1,000, respectively. Secondary fluorescent Alexa Fluor antibodies, goat α-rabbit 568 and goat α-mouse 488 (ThermoFisher), were used at a 1:1,000 dilution. Images were acquired using a Leica SP5 MP confocal laser-scanning microscope, processed by deconvolution using Huygens software, and visualized with ImageJ software.

### Generation of transgenic parasites.

Targeted disruption of P. berghei *PIMMS2* was achieved by double-crossover homologous recombination in the *c507* line as previously described (51). Briefly, a fragment of 591 bp, extending from the 287-bp position of the 5′ untranslated region (UTR) to the 304-bp position in the coding region, and a fragment of 535 bp of the 3′UTR of *PIMMS2* were amplified from P. berghei genomic DNA using the primers P1, P2, P3, and P4, which contained appropriate restriction enzyme sites (Table S4). They were then cloned into the pBS-TgDHFR vector that carries the modified Toxoplasma gondii dihydrofolate gene (Tg*DHFR/TS*), which confers resistance to pyrimethamine (4).

To generate Δ*pbpimms2*::*pimms2*^*wt*^ and Δ*pbpimms2*::*pimms2*^*mut*^ transgenic parasites, the ORF of *PbPIMMS2*, including the 5′UTR and a region downstream of the Δ*pbpimms2* locus, was amplified from P. berghei genomic DNA using primers P8-P9 and P10-P11, respectively (Table S4). These regions were then cloned into the PL0035 vector that carries the positive selectable marker human dihydrofolate reductase (DHFR), which confers resistance to WR92210 (53). For the P. berghei PIMMS2 mutagenized complement construct, site-directed mutagenesis of the predicted catalytic residues was carried out using the forward primers, P13-P15 (Table S4), and the QuikChange multisite-directed mutagenesis kit according to the manufacturer's guidelines (Agilent). Transfection of linearized targeting construct, selection of transgenic parasites, and clonal selection were carried out as described previously (51).

### Genotypic analysis of transgenic parasites.

Transfected blood-stage cell populations were obtained, and white blood cells were removed using CF-11 columns (Whatman). Red blood cells were lysed by incubation in 0.17 M ammonium chloride on ice for 20 min, and genomic DNA was extracted from parasites using the DNeasy kit (Qiagen). Detection of successful integration events was performed by PCR on genomic DNA using primers listed in Table S4. Southern blot analysis on pulsed-field gel electrophoresis-separated chromosomes was carried out with a probe containing the HindIII/EcoRV fragment of *TgDHFR/TS* obtained from the pBS-TgDHFR plasmid.

### Exflagellation assays.

Parasite-infected blood was mixed in equal parts with RPMI 1640 (Life Technologies) supplemented with xanthurenic acid and hypoxanthine. Following 10 min of incubation at 21°C, the number of exflagellation centers was counted. The number of exflagellation centers was then compared to the number of male gametocytemia determined from Giemsa-stained blood smears.

### Macrogamete-to-ookinete conversion assays.

One hundred microliters of a 24-h *in vitro* ookinete culture was incubated with a Cy3-labeled P28 antibody (1:50 dilution) for 10 min on ice. The conversion rate was determined as the percentage of Cy3-positive ookinetes to Cy3-positive parasites (macrogametes and ookinetes).

### Ookinete invasion assay.

For midgut invasion assays, A. gambiae L3-5 mosquitoes (54) were infected with P. berghei by ookinete membrane feeding. Melanized parasites present on dissected midguts were visualized 7 dpi under light microscopy.

### Ookinete motility assays.

Ookinete motility assays were performed as previously described (55). Briefly, ookinete cultures were added to an equal volume of Matrigel (BD) on ice, mixed thoroughly, dropped onto a slide, covered with a Vaseline-rimmed coverslip, and sealed with nail varnish. The Matrigel was then allowed to set at room temperature for at least 30 min. After identifying a field containing ookinetes, time-lapse microscopy images (1 frame every 5 s for 10 min) of ookinetes were taken using the differential interference contrast settings with a 636 objective lens on a Leica DMR fluorescence microscope and a Zeiss AxioCam HRc camera controlled by the AxioVision (Zeiss) software package. The speed of motility of individual ookinetes was measured by multiplying the number of body lengths moved by the length of the ookinete during the 10-min video and divided by 10. Multiple independent slides and cultures were used for each parasite line. Video processing and annotations were carried out using the AxioVision or AxioVision LE (Zeiss) software.

### Statistics.

For statistical analyses of the oocyst load (infection intensity) and presence of oocysts or melanized parasites (prevalence), *P* values were calculated using the Mann-Whitney *U* test and the chi-squared goodness-of-fit test, respectively. Statistical analysis for exflagellation, ookinete conversion, and motility assays was performed using a two-tailed, unpaired Student *t* test.

### Imaging and enumeration of parasites.

Following mosquito infection and dissection, midguts were fixed in 4% (vol/vol) formaldehyde (16% methanol-free, ultrapure stock diluted in PBS; Polysciences Inc.) for 20 min at room temperature and washed three times for 10 min in PBS. Fixed midguts were mounted in Vectashield (Vector Laboratories) on glass slides under sealed coverslips. Oocyst numbers were counted at 10 dpi using fluorescence microscopy under ×10 magnification. Midgut and salivary gland sporozoite numbers were counted using a hemocytometer from homogenates of 10 P. berghei-infected A. gambiae or A. stephensi midguts or salivary glands at day 15 or 21 dpi, respectively. The numbers of sporozoites per mosquito were calculated.

### Transmission from mosquito to mouse.

P. berghei-infected mosquitoes were fed on anesthetized C57/BL6 mice at 18 and 21 dpi. Parasitemia was monitored on days 5, 7, 10, and 14 postinfection by Giemsa-stained tail blood smears.

### Homology modeling.

Homology modeling was carried out using the homology/analogy recognition engine Phyre^2^ (12). The PIMMS2 homology model was generated based on the highest-scoring template (PDB code 4TR2; 100% confidence), corresponding to P. vivax subtilisin 1 (PvSUB1). All structural figures were generated using the PyMol Molecular Graphics System, version 1.3 (http://www.pymol.org/; Schrödinger LLC, Portland, USA).

### Bioinformatics analysis.

Plasmodium sequences (DNA and protein) were retrieved from PlasmoDB, T. gondii sequences were retrieved from ToxoDB, and Cryptosporidium parvum and Neospora caninum sequences were retrieved from NCBI. The presence of a putative signal peptide and transmembrane domain was predicted using Phyre2 (12), TargetP (14), and Phobius and PolyPhobius (13). Sequence alignment was carried out using ClustalW2. The BioEdit Sequence Alignment Editor was used for visualization of the alignment.

## Supplementary Material

Supplemental material
