# Supplementary material for: Plasmodium berghei PIMMS2 Promotes Ookinete Invasion of the Anopheles gambiae Mosquito Midgut
Source: Infect Immun. 2017 Jul 19;85(8):e00139-17. doi: 10.1128/IAI.00139-17 (PMC5520436; doi:10.1128/IAI.00139-17)
Supplement: Supplemental material [file supp_85_8_e00139-17__index.html]

Supplemental material 

# Plasmodium berghei PIMMS2 Promotes Ookinete Invasion of the Anopheles gambiae Mosquito Midgut

## Supplemental material

- Supplemental file 1 -

  Fig. S1. PIMMS2 3-D homology model. Fig. S2. Multiple sequence alignment of PbPIMMS2, PvSUB1, and bacterial BPN′. Fig. S3. Multiple sequence alignment of *Plasmodium* PIMMS2 orthologs. Fig. S4. Generation and genotypic analysis of Δ*pbPIMMS2* mutant parasites. Table S1. Effect of *PIMMS2* knockout on oocyst development in *A. gambiae* and *A. stephensi*. Table S2. Effect of *PIMMS2* disruption on sporozoites development and mosquito-to-mouse transmission. Table S3. *PIMMS2* genetic complementation analysis. Table S4. Primers for RT-PCR, generation of transgenic parasites, and protein expression.

  PDF, 882K
