# Supplementary material for: Plasmodium berghei PIMMS2 Promotes Ookinete Invasion of the Anopheles gambiae Mosquito Midgut
Source: Infect Immun. 2017 Jul 19;85(8):e00139-17. doi: 10.1128/IAI.00139-17 (PMC5520436; doi:10.1128/IAI.00139-17)
Supplement: Supplemental material [file IAI.00139-17_zii999092107s1.pdf]

**Fig. S1**

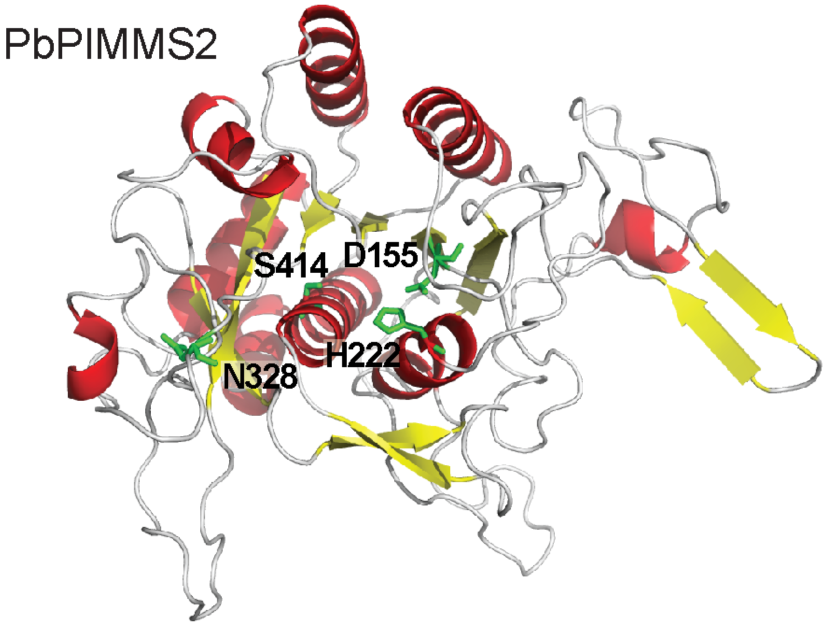


**FIG S1** PIMMS2 3D homology model. A 3D homology model of the subtilisin-like domain of PbPIMMS2 based on the known structure of PvSUB1. Conserved amino acid residues of PbPIMMS2/PvSUB1 D155/316, H222/372, N328/453 and S414/538 are shown in stick form.

# Fig. S2

```
PbPIMMS2    1    MVLLN-------GKLKYIAVVAIFYNLIIILLVKEKFPYICTKKKFHAISNRILYEYLNN- 52
PvSUB1      1    MVLTR-149aa-GKLSNLKNLKS---MIIDLNS-------------DMTDEELAEYISLL 186
BPN’        1    MR----------GK---------------------------------------KVWISLL 11
Consensus        *           **                                         ::.

PbPIMMS2    53   ------FVSKDIFRREDITLKN-------------LNFVQT--NLK--------S----- 78
PvSUB1      187  ERKGALIESDKLVGADDVSLASVKDAVRRGES--SVNWGKLRSTMLEVPSGESPPSHAAS 244
BPN’        12   FALALIFTM--AFGSTSSAQA---AGKSNGEKKYIVGFKQTMSTMSAAKKKDVISEKGGK 66
Consensus              :     .   . :                ; : :   .:

PbPIMMS2    79   ---------DK-----DAEIKEN---RDTQSVDDNMFQRVYKFILNFFYGNKKNRINKSM 121
PvSUB1      245  SGSPFD---DDDDLLSEAALHRE-----EAHLAGSKTTKGYKFNDE----------YRNL 286
BPN’        67   VQKQFKYVDAASATLNEKAVKELKKDPSVAYVEEDHVAHAYA---------------QSV 111
Consensus                        :  ::.         :  .   : *                :.:

PbPIMMS2    122  NYGKYDNFNKINDIFEFMRNNGLPINITSVCLIDTGLNIKDALINYFLNHDISTYNSYTY 181
PvSUB1      287  QWGLDL--ARLD--ETQDLINANRVSVTKICVIDSGIDYNHPDLRNNIDVNVKELHGRK- 341
BPN’        112  PYGVSQ--IKA----PALHSQGYTGSNVKVAVIDSGIDSSHPDLKVAGG----------- 154
Consensus         :*      :          :.   . ..:.:**:*:: ..  :.

PbPIMMS2    182  HSVNINYKKPDSFNFGINSENCDEDNYSECESTFLENHNGHGKYED--KSTIQGDSLKLI 239
PvSUB1      342  -GV-------DDDSNGVVDDVY-GANFVNNSGDP-MDDNYHGTHVSGIISAVGNNGIGIV 391
BPN’        155  ------------------------ASMVPSETNPFQDNNSHGTHVAGTVAAL-NNSIGVL 189
Consensus                                 .    .     :.* **.:     :::  :.: ::

PbPIMMS2    240  EKKYDKNVDLQRSGIDVEICKAFNNSKEKKNSLNIIPVIKCLEYCKTKNVKIIHMDYNIN 299
PvSUB1      392  G---------VDGHSKLVICKALDQHKLGRL-GDMAHMINGS-FSFDEYSNI-------- 432
BPN’        190  G---------VAPSASLYAVKVLGADGSGQY----SWIINGIEWAIANNMDVINMSLGGP 236
Consensus                       .:   *.:      :       :*:   :.  :  .:

PbPIMMS2    300  EQNEQLIQIMDDLKNSEIFVILPSEKLFNEK---P-YE-------DNSVIYPSSFFEKFE 348
PvSUB1      433  -----FNASVEHLRSLGILFFVSA---SNCAHDKLSKPDIAKCDLAVNHRYPPILSKTHN 484
BPN’        237  SGSAALKAAVDKAVASGVVVVAAA---GNEGTS------GSSSTVGYPGKYPSVIA---- 283
Consensus             :   ::.     :...  : :  *.                    **  :

PbPIMMS2    349  NVFFIGSLDYSDMSSDDADIASNFQIQKN--EYLKYRKNNVFLLDSINSSLKKRDDHDIL 406
PvSUB1      485  NVIAVANLKRDLD--------ESYSLSVNSFYSNIYCQLAAPGT----NIYSTTPM--NN 530
BPN’        284  ----VGAVDSSNQR-------ASFS------SVGPELDVMAPGV----SIQSTLPG--NK 320
Consensus            :. :. .           .:.            .     .       .:     :

PbPIMMS2    407  YYEIKYSS---AFFINIITIILNIYPNMSIKELRNILSYSIPSKETAQLKTENIFEG-NF 462
PvSUB1      531  YRKLNGTSMASPHVAAIASIVRSINPNLTYLQIVEILRNAIVKLPS--LTERVSWGG-YV 587
BPN’        321  YGAYNGTSMASPHVAGAAALILSKHPNWTNTQVRSSLENTTTKLGD-----SFYYGKGLI 375
Consensus        *     :*: :..     ::: .  ** :  :: . *  :  .           :    .

PbPIMMS2    463  DINKFIHILLNRGINSSGFVRKYKDVTPNESTN-340aa-R 836
PvSUB1      588  DILRAVNLAIDS--KAAPYIKSHSWFRWKQGSRR------- 619
BPN’        376  NVQAAAQ---------------------------------- 382
Consensus        ::    .
```

**FIG S2** Multiple sequence alignment of PbPIMMS2, PvSUB1 and bacterial BPN'. The conserved amino acid residues forming the putative catalytic triad (D155, H222 and S414) and stabilizing of the oxyanion hole (N328) are marked in red. Regions with no homology are shown as number of amino acids (aa). Black and gray shaded residues indicate conservation and similarity, respectively. BPN', Uniprot: P00782 and PvSUB1, PVX_097935.

## Fig. S3

```
PbPIMMS2    1    MVLLNGKLKYIAVVAIFYNLIIILVVKEKFPY--ICTK---KKFHAISNRILYEYLNNFV- 54
PyPIMMS2    1    MVLLNGKFKYIAVVAIFYNLIIILVVKEKFPY--MCTQ---QKSHTISNRILYEYLNNFV- 54
PcPIMMS2    1    MVLLNGKRKYIAVVAIFYSFIILLVKEKFPY--ICTQ---KKSHTINNRILYEYLNNFV- 54
PkPIMMS2    1    MLFTKERHRRVAVSTIFFHIIALLQFSGDATIHWCRRGSERVQRAMNGRLLEEGQIGKI- 59
PvPIMMS2    1    MPFTKERQRHVVVATIFCHLAALLLFGGDATVHRCRRGSEPLQRTVNGRVLLEGQVGSL- 59
PfPIMMS2    1    MIYLGKKLLSCTLFVYFLYIHFFLLKQ----NNFCDV-------KVRERILEESINNDLS 49
Consensus        *      :     .: .  *  :   :*          *             :   *:*  *      :

PbPIMMS2    54   --SKDIFRREDITLKNLNFVQTNLKSDK-DAEI----KENRDT----------------- 90
PyPIMMS2    54   --SKDIFRRDDITLKNLNFVETNLKNDK-DAEI----KENSDT----------------- 90
PcPIMMS2    54   --SKDIFRREDITLNNLNFVQTNLKYDK-DVEI----KENNDA----------------- 90
PkPIMMS2    59   --PNVLHNYSKMNLSTKSFVQVKVKDSH-DRNI----RRGGVTTSEGNYHIP----NCDD 108
PvPIMMS2    59   --ADELHNYSKTNLSIPSFVQVKLKDSY-DRKI----RRGGA-TPEGNYHIP----NCDD 107
PfPIMMS2    50   SKGENLHIYEKTNVSVQTFIKKKERKNLSDNNINDKINNNNNYNNNNNNNIEDTTYYPTG 109
Consensus          . :.   .. .:.   .*:: : :  .   *  :*     ..

PbPIMMS2    91   QSVDDNMFQRVYKFILNFFYGNKKNRINKSMNYGKYDNFNKINDIFEFMRNNGLPINITS 150
PyPIMMS2    91   QSVDDNMFQRVYKFVLNFFYGNKKNRINKSMNYGRYDNFNKINDIFEFMRNNGLPINITS 150
PcPIMMS2    91   QNADDNIFQRMYKFVLNFFYENKKDSTNKYMKYGKYEHFNKINDIFEFMRNNGLPINITS 150
PkPIMMS2    109  TDESSNFLKKFCRSMKSFVGI-TPHD---KDWCLKYEKFRRFKQHFVYMVQNNLSLGKTR 164
PvPIMMS2    108  TDGSANFLKKMYRSIKAFLRI-TSHE---KSWCLKYERFRRVRRDFVYVVQSSLSVGRTR 163
PfPIMMS2    110  NEKKENIFLKIFRYVKNWFPIKSSNNL--KKTNINYEQVQEENEFSKYILQNNMSIETTK 167
Consensus         .  . *::  :.  :  :   :.    .  .           .*:.... .     ::  :.  :  :   *

PbPIMMS2    151  VCLIDTGLNIKDALINYFLNHDISTYNSYT-------------YH-----------SVNI 186
PyPIMMS2    151  VCLIDTGLNIKDELINYFLNHDISTYNSYT-------------YH-----------SVNI 186
PcPIMMS2    151  VCLIDTGLDIKDEIINHFLNNDISTYNSDT-------------FH-----------SVNI 186
PkPIMMS2    165  VCLIDTGLDLQDKVVRQFVKIYRWEHSKEGDNPSERSGGGEYDTHLQLEERDDEGASPSW 224
PvPIMMS2    164  VCLIDTGVDLKDEVLGHFVRMSRGGLNQGGDNPGEQ------------------------ 199
PfPIMMS2    168  VCLIGSGGDSSHDLIKQFLLHNNVKYNRNYENDNSLNNKS------------GSIISSYK 215
Consensus        **** :* :  ..  ::   *:          .

PbPIMMS2    187  NY-KKPDSFNFGINSENCDEDNYSECESTFLENHNGHGKYEDKSTIQGDSLKLIEKKYDK 245
PyPIMMS2    187  NY-KKPDSFNLGVNSENCDEDSNSECESTFLENHNGYEKYEDRSKIQGDTLKLIEKKYEK 245
PcPIMMS2    187  NY-KNADSFNFEINTENRDEDDYPEYESTFLENHNGDGSYEANSTSQGDPLKWGEKKYKK 245
PkPIMMS2    225  AYPKGGDAKYDGINTERCNEENYARCQSSDIDDVDMHGTFIANTVIRRDLLMKR-EMYKK 283
PvPIMMS2    199  -----NERRADGINTQRCNEKNYASCQSSDVHDEDMHGTFIANTVIRRDLLMKG-GAYKR 253
PfPIMMS2    216  YYSEEIIQEPYIIDRDICNKK--KNCKESTLNYENNPKTLIGNIIIQSDILKNE-KIFNM 272
Consensus                    :: :  ::.      :.: :.   :    .    .    : *  *         :.

PbPIMMS2    246  NVDLQRSGIDVEICKAFNNSKE-K---KNSLNIIPVIKCLEYCKTKNVKIIHMDYNINEQ 301
PyPIMMS2    246  NVDLQKSGINVEICKTFDNSKE-K---KNDSNIIPVIKCLEYCKTKNVKIIHIDYNINEK 301
PcPIMMS2    246  GMDLQRSGIHVETCKAFDNSKE-S---KNGSNIIPIIKCLEYCKTKNVKIIHIDYNINEK 301
PkPIMMS2    283  -------NVELIVCKAFGDREE------TNSHLMPLIKCLEHCKGSGAKVIHVGYNVEGE 330
PvPIMMS2    253  -------GVDLIVCNAFAKSFGNA---VKNSHLVPLIKCLEMCKERGAKVIHVGYNVQGE 303
PfPIMMS2    272  -------NRHFVVCKYFGSIPKTNIKINQSTLIQHLIKCLDYCKMEGVQYIYIGYNIYAA 325
Consensus                   ..   *: *  .              :   :****:  **    .:  *::  **:

PbPIMMS2    302  NEQLIQIMDDLKNSEIFVILPSEKLF--NEKPYED------------------------- 334
PyPIMMS2    302  NEQLMQIIEDLKKSEIFVVLPSKKLF--NEKSHGD------------------------- 334
PcPIMMS2    302  NEELIEIIEDLKNSEIFLLLPSKGLL--NEKSYED------------------------- 334
PkPIMMS2    331  SEKLVEVMQELERAQIVVVSPSLRVYTGQSDESQQPR---------------------KE 369
PvPIMMS2    304  SEQLVKLMEELQREEIIVVSPSLQVYHRNGGETNSK----------------------KE 341
PfPIMMS2    326  NNKLIEIMKKLREHKIIIVTSSGKIYDDDNNNDNNNFYNDNIYNNNIYNVHNDDKEKIKN 385
Consensus        .::*::::..*..  :*.::   *   :.   .

PbPIMMS2    334  ---------------------NSVIYPSSFFEKFENVFFIGSLDYSDMSSDDAD---IA- 369
PyPIMMS2    334  ---------------------NSVIYPFPFFEDFEHFEKFE------------------- 354
PcPIMMS2    334  ---------------------NSVAYPSSFFENSENVFFIGSLNYLDVFSYDVN---IA- 369
PkPIMMS2    370  HGEEH---------------STEKLYPSSFADTFENVFSVGALRNSTQGGFVPISGNGN- 413
PvPIMMS2    342  HLEEP---------------STQKMYPASFADTFENVFSVGALRNSPQGGLVPILGNAN- 385
PfPIMMS2    386  HIKKKNKQNNYLYEYQRTQKDEEQKSNSSLYQNLENVISISGLIYTDSSKKNKNKNYIYD 445
Consensus                              .      : :   *..  .
```

```
PbPIMMS2     369  --SNFQIQKNEYLKYRKNNVFLLDSINSSLKK-RDDHDILYYEIKYSSAFFINIITIILN 426
PyPIMMS2     354  ---------------KFENVFFLDSINSSLKK-GDDHDMLDYEIRYSSAFFINIITIILN 398
PcPIMMS2     369  --SNSQAQKNEYLKYRKDNVFLFDSINGSLKK-GNDHDISYYEVRYSSAFFINILTTILN 426
PkPIMMS2     413  --PKGEKQKWKVLHKRENTTLFSFSYGKTFPFGRSPSSMVEDGEGYASADFVNILVMILN 471
PvPIMMS2     385  --PRGEKPKGEQLHKRENTTLFSFSYGKTFPFGRSPPSMVEDAQAYASADFVNALVMIFN 443
PfPIMMS2     446  NEIKILDQKGNKKLNRNYISLFYFSYDTDIYE-KIESDIIDDDHDLVSASFVNTLVLMHS 504
Consensus                        :    ::  *    :       .:        ** *:* :. : .

PbPIMMS2     427  IYPNMSIKELRNILSYSIPSKETAQLKTENIFEGNFDINKFIHILLN------------- 473
PyPIMMS2     399  IYPNISIKELRNILSYSILSKETPELKTENIFEGNFDINRFIHILLN------------- 445
PcPIMMS2     427  VYPNISINELRNMLSYSIIPEETDELKSQDIFEGNFEINKFIHGLLN------------- 473
PkPIMMS2     472  VIPKLSIRRMRHILKRSIVKRS--EMKGLSKWGGYIDPLKVIDATLKERNELCKTFFGEL 529
PvPIMMS2     444  VNPKLSMKRVRLILERSIGRRS--ELKGLSKWGGYLDPFKLIAETLKERNELCGRFFREL 501
PfPIMMS2     505  INLKLSLGRLRKILNKSIVKRE--ELRHLSNRAYYHDMMNTFEDSLNQRKRSYKIFYLEL 562
Consensus         :  ::*: .:* :*. **  ..  :::  .      :  . :   *:

PbPIMMS2     473  -------------------RGINSSGFVRKYKD---VTPNESTNKIFLLEDQKDADIEPQ 511
PyPIMMS2     445  -------------------RGIIYSDFVQKYED---VTPNEPTNKTSVLEGQEDGEIEPQ 483
PcPIMMS2     473  -------------------GETNSSEYVRKSKD---VPSKEFTNKEFVSEDQEDDIVEAE 511
PkPIMMS2     530  DLDLEAEGGSSSFRGDLSKGGFSGGSMTEDLGDWVEKPTTGTEEQIF--DKTITGDVTPR 587
PvPIMMS2     502  GGNLEEGGG------GLLGGGLPRGEATEHLGDWVERPPNDEDQRSC--GEATTGEATPR 553
PfPIMMS2     563  KNNKHKVLL-------------SDANLKSMYQDNLPVNYNEED--------HVKHNVEQE 601
Consensus                                 .       *      .                   .

PbPIMMS2     512  KDSSIDIYCDEGG-------------------------------------------SD 526
PyPIMMS2     484  TDSSTDIYYDEDGSGEGGS---DEGGS---GEG-GSGEGGSGEGGSG---EGGSGEGGSG 533
PcPIMMS2     512  VEVEAEAEVEAEAEVEAEV---EAEAE---VEAEVEAEVEAEADSSA---DADYDKDGLD 562
PkPIMMS2     588  LNDEQEESEKATGHLEDQVIFPQQRGEMEEMERHLVGETLNGLDPEERNDLGRYDMAHYD 647
PvPIMMS2     554  LD---------------AAFPPQGGELEEIERQFEEEAVRGLDH--------HNLGYYH 589
PfPIMMS2     602  TSVERDIYKNNENSNKNRK------MDMDEG-----------------------KGTYI 631
Consensus          .

PbPIMMS2     527  EDIVSTSNGLDVYSEYSHSNNKSDQLLNDEKGLK------------YETYKDLYSVKEND 574
PyPIMMS2     534  EDVLSTLNELDDYSEDSYSNNKSEQLLEDENGLEDEPLLEDENEFEGETSKDLYSVKEND 593
PcPIMMS2     563  EDPINETDELDEPNEDSYEDNQSEKLLEDEN-----------YEFEEDPSKDLHSVKEND 611
PkPIMMS2     648  MDVISKWE---------------EDFSDGVAALREDPASDTTFTSMYNYEENVYVPDEAK 692
PvPIMMS2     590  TDAISKWA---------------EDFPDGGAAPSEGPPSDTTYRSSYNDEEDVYTPDEAN 634
PfPIMMS2     632  QNKES-----------------------------------HKYNIHYPY-------NRIK 649
Consensus         :  .                                                   .. .

PbPIMMS2     575  IYVF---------------ESNNPTNSSFMQMNYDDK---IKSKYLDNLEEANYQNHRTQ 616
PyPIMMS2     594  IYVI---------------ESKNPTNSSFMQMNYDDR---IKSKYVDNLEDANYQNHRAQ 635
PcPIMMS2     612  IYVL---------------ENENPFNSSFMQISYDDK---VRPKYVDNLDEGNYRP---Q 650
PkPIMMS2     693  ESFYEDAGRVTV----TGEMSSLPLGFSFLENHTNDRGSDLP-LYRTNERGQVYASGDGT 747
PvPIMMS2     635  QSFYGNSGGVALEVVGEGGLSSLPLGVSFLDKHTSDGGSAVPPLPRRNGRGRGYESGEGT 694
PfPIMMS2     650  QSLL-----------NDNTLNHKP-YVSFLNMSYYNED----------IEKRNYNIYDDP 687
Consensus           .                 .  *   **::    :                 *

PbPIMMS2     616  --FEINRN----DNRYPI-----------IS---ED--N---LRDRQNMHNIQMLNDGIN 651
PyPIMMS2     635  --FEINRN----GNRYPI-----------FS---ED--N---LRDRQNTHNIQMVNDGIN 670
PcPIMMS2     650  --Y----E----MNRYPI-----------IS---GD--S---LRGRQNMNNIQMVNDDIH 681
PkPIMMS2     748  PGLPLNRR-SQDENGFPERWGQQGRQ-AMIEEDSGE--G---YHHGQDDWVTQMKG--TK 798
PvPIMMS2     695  S-PPMNRR-SLGESGLPERWDQTEGTHVMNEEDRGD--G---Y-DGQADWITQMRG--TN 744
PfPIMMS2     687  -SYTYDQGITYDDNYYIDDHDIHTRKKRKISYDGEDNNDYHMYDDRDNLFHSNLGN--NK 744
Consensus                      .                .    :          :     ::     .

PbPIMMS2     652  YS-ENANNIEELYDNDFEDYRGKDLNPEYNKIRDNNNNKNINKEFGILNTWRKNNEGLYL 710
PyPIMMS2     671  YS-ENGRNIEELYDNDFEGYRGKNLNPEYNNN--NNNNNNINREFGILNSWRKNDEGLYL 727
PcPIMMS2     682  YS-ENANNIEELYDNNFEGYRGNNLNQEYNKNYD--NNKNVNSEFGGLNNWRRNNEGSYL 738
PkPIMMS2     799  SVDDPEYDVGRMYRDSPEGIPAEELLR-----------SGWRGTTPPLSRWNRNGETSL- 846
PvPIMMS2     745  YVDGHAYKAARVYGNSPEGIPEGELSR-----------PDWGWPTTPLSRWDPRGETPP- 792
PfPIMMS2     745  YEDDGNV----------HREKEKDLEPR--------------FLYDPFANIENRDLE--- 777
Consensus                          .     :*                      :     .

PbPIMMS2     711  SDSEEESQPFKWMDMHVDDIYQDRKKRLKGNNYDNRNGRVRR-----IYEDNKRGRYIKN 765
PyPIMMS2     728  NDNEEASQPFEWMDMHADDIYQDRKKRLKENNYDNKNGRGRR-----IDEDNKRGRYIKN 782
PcPIMMS2     739  NDSEEVHHPFEWMDMQAGDIYRDKKNRLKENNYDNNETEIRRRRKRGRGGENKRGRSMKN 798
PkPIMMS2     846  -----------WGDENEGYVFSPN----VDNNWGEKDGRNRRN----------------- 874
PvPIMMS2     792  -----------WGDDHEGYGFSPQ----LDDDWGGEYGRKGRRRDE-------LGRRLRR 830
PfPIMMS2     777  ----TVQELSELREKKSNNFYSR--------NHDNSSNMKRRR-KEKK---KKLKKVLRS 821
Consensus                      : .    :          :         *
```

```
PbPIMMS2        766  RNM----------QKKKNFDRNLKN--KRYLNRR-TKRHINEKNKRIMREKKNKYNNSVL 812
PyPIMMS2        783  RNM----------QKKKKFDRDVKN--KRYLNRRRSKRQISKKNKRIMREKKNKYNNSIL 830
PcPIMMS2        799  RNM----------RKTKKLGRDVKN--RRYINRRT-KRQI---------DKENKYNNSAL 836
PkPIMMS2        874  --PQRRRSRDDLERRRRRFPRAMPRQRRRIPRRGRSKRRLKQREAGVVRK--NGRNGKRR 930
PvPIMMS2        831  DELGRKRTRDELRRRRRRSPRAVPRQRRRTPTRGRTAKRLTGREAGLVRK--NGPTGKSR 888
PfPIMMS2        822  K-Y---DKIGNLERIRRKKKRRMIHK-NKINKRRNMKRK----NNE-L--------EERR 863
Consensus                         :  :.  * : .  .:   *   ::                   .

PbPIMMS2        813  KRNEMKSHN---------------------------------------NSQKTPKIIPRK 833
PyPIMMS2        831  KRNEMKSHN---------------------------------------NPQKSPKIIPRR 851
PcPIMMS2        837  KRQEMKSYN---------------------------------------NSQKAPKIIPRR 857
PkPIMMS2        931  NENGMRSRREGEY---------------------SNMPMRRRPVRGKPVPGFAPKMPRVV 969
PvPIMMS2        889  NGNAMRFRSSGRSVRGRSQRRRPVGGRPVLRKPLLRKPLLRKPILGMPVRAFAPKTSRVV 948
PfPIMMS2        864  NKQADKNSSSGNG-KGK------ING-----------------------TRNSPKIKFK- 892
Consensus            : :  :                                              :**

PbPIMMS2        834  YSR- 836
PyPIMMS2        852  YSR- 854
PcPIMMS2        858  YSR- 860
PkPIMMS2        970  MGRR 973
PvPIMMS2        949  MGRR 952
PfPIMMS2        892  --R- 893
Consensus              *
```

**FIG S3** Multiple sequence alignment of *Plasmodium* PIMMS2 orthologs. Sequence alignment of PIMMS2 orthologs in six *Plasmodium* species: *P. berghei* (PbPIMMS2; PBANKA_1106900), *P. yoelii* (PyPIMMS2; PYYM_1109100), *P. chabaudi* (PcPIMMS2; PCHAS_1106600), *P. knowlesi* (PkPIMMS2; PKNH_1026300), *P. vivax* (PvPIMMS2; PVX_097925) and *P. falciparum* (PfPIMMS2; PF3D7_0507300). The residues forming the putative catalytic triad (Asp, His and Ser) and the oxyanion hole-stabilizing residue (Asn) are highlighted in red**.** Black and gray highlighted residues indicate identical and conserved amino acids, respectively.

**Fig. S4**

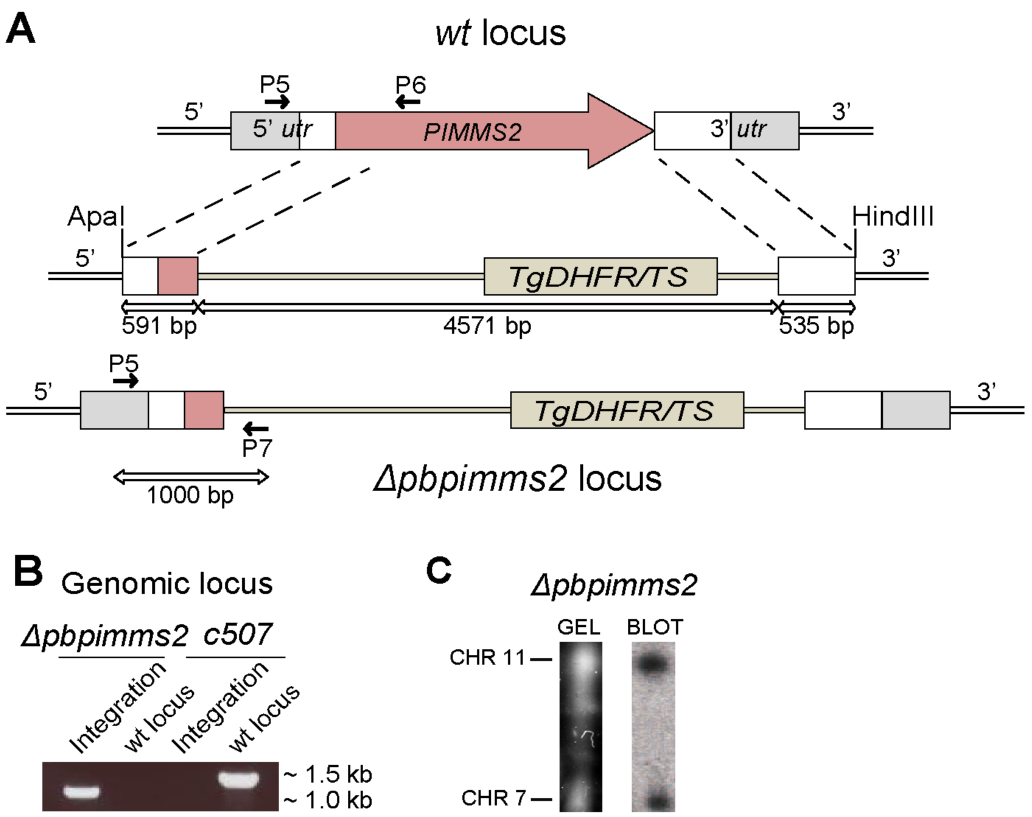


**FIG S4** Generation and genotypic analysis of *ΔpbPIMMS2* mutant parasites. (A) Schematic representation of the native *PIMMS2* (*wt*) and the modified *Δpbpimms2* genetic loci. The disruption vector carries gene targeting sequences for *PIMMS2,* which flank the pyrimethamine based selectable marker *TgDHFR/TS*. The pBS-TgDHFR/TS gene disruption vector is presented in the middle. Integration of the ApaI/BamHI linearized vector results in replacement of *PIMMS2*. (B) Genotyping of *Δpbpimms2* mutant parasites by PCR based analysis of the genomic DNA from *c507* and cloned parasites show that the *Δpbpimms2* locus is only present in the knockout line. (C) Southern blot analysis on pulse field electrophoresis separated chromosomes from cloned *Δpbpimms2* parasites. The *dhfr/ts* cassette is detected at chromosome 11 corroborating integration of the disruption vector.

**Table S1**. Effect of *PIMMS2* knockout on oocyst development in *A. gambiae* and *A. stephensi*

| Time | Parasite | Number of exp | Number of midguts | Prevalence (%) | Infection intensity | | Infection range | P-value | Fold difference |
|---|---|---|---|---|---|---|---|---|---|
| | | | | | Arithmetic mean | Median | | | |
| Day 10 | *A. gambiae* infections | | | | | | | | |
| | *wt* | 4 | 102 | 89.2 | 61.5 | 38 | 0-368 | | |
| | *Δpbpimms2* | 4 | 117 | 82 | 5.8 | 3 | 0-38 | <0.0001 | -11 (-13) |
| Day 10 | *A. stephensi* infections | | | | | | | | |
| | *wt* | 2 | 39 | 100 | 495.4 | 503 | 14-902 | | |
| | *Δpbpimms2* | 2 | 60 | 98 | 101 | 93 | 0-281 | <0.0001 | -5 (-5) |
| Day 10 | *A. gambiae L3-5* infections | | | | | | | | |
| | *wt* | 2 | 31 | 100 | 30.2 | 23 | 0-91 | | |
| | *Δpbpimms2* | 2 | 42 | 57 | 5.2 | 2 | 0-29 | <0.0001 | -6 (-12) |

The table reports results from *Δpbpimms2* or control *c507* (*wt*) parasite infections of *A.gambiae*. *Δpbpimms2* or *wt* infected midguts from independent biological replicates (indicated in the third column) were pooled for each group. The total number of midguts is indicated in the fourth column. Midguts with zero parasites were also considered for calculation of the arithmetic means of parasite densities. Prevalence shows the percentage of midguts with at least one oocyst. *P* values were calculated using the Mann-Whitney *U*-test. Fold differences between *Δpbpimms2* and *wt* oocyst densities were computed using both the arithmetic mean and the median (in parenthesis). In L3-5 infections, the density data refers to the number of melanised ookinetes.

**Table S2**. Effect of *PIMMS2* disruption on sporozoites development and mosquito-to-mouse transmission

| Parasite | MG sporozoites | | SG sporozoites | | MG/SG | Infectivity to mice | |
|---|---|---|---|---|---|---|---|
| | Mean | SE | Mean | SE | | Day 18 | Day 21 |
| *A. stephensi* | | | | | | | |
| *wt* | 71,133 | 5,452 | 7,660 | 2,548 | 9.3 | 2/2 | 2/2 |
| | 57,612 | | 16,930 | | 3.4 | 2/2 | 2/2 |
| | 48,120 | | 7,476 | | 6.4 | 2/2 | 2/2 |
| *Δpbpimms2* | 8,854 | 570 | 2,057 | 389 | 4.3 | 2/2 | 2/2 |
| | 10,180 | | 3,650 | | 2.8 | 1/2 | 2/2 |
| | 11,270 | | 3,229 | | 3.5 | 2/2 | 2/2 |
| *A. gambiae* | | | | | | | |
| *wt* | 11,225 | 2,929 | 1,695 | 508 | 6.6 | 1/2 | 2/2 |
| | 23,216 | | 1,897 | | 12.2 | 1/2 | 2/2 |
| | 14,400 | | 3,656 | | 3.9 | 1/2 | 2/2 |
| *Δpbpimms2* | 1,800 | 340 | 325 | 190 | 5.5 | 0/2 | 0/2 |
| | 3,220 | | 517 | | 6.2 | 1/2 | 1/2 |
| | 2,728 | | 1,100 | | 2.5 | 1/2 | 1/2 |

The table outlines mean of *Δpbpimms2* or *wt* sporozoite densities in *A. stephensi* and *A. gambiae* midguts (MG) and salivary glands (SG). The mean was calculated by quantifying the number of sporozoites in suspensions from three pools of ten homogenised midguts or salivary glands, respectively, at day 21 post infection. SE represents standard error. The ratios of midgut to salivary gland sporozoites are shown. Infectivity to mice was assayed by allowing *Δpbpimms2* or *wt* infected mosquitoes to feed on two C57BL/6 mice (bite-back) at day 18 and 21 of infection, respectively. Mice were allowed to recover and parasitaemia was assessed at day 5 post-feeding and up to day 14 if infection was not detected.

**Table S3**. *PIMMS2* genetic complementation analysis

| Experiment series | Parasite | Number of exp | Number of midguts | Prevalence (%) | Infection density | | Parasite range | *P*-value |
|---|---|---|---|---|---|---|---|---|
| | | | | | Arithmetic mean | Median | | |
| 1 (Fig. 4D) | *Δpbpimms2*::*pimms2*$^{wt}$ | 2 | 50 | 82 | 26.2 | 14 | 0-146 | |
| | *Δpbpimms2* | 2 | 102 | 79 | 6.3 | 3 | 0-120 | <0.0001 |
| | *wt* | 2 | 59 | 90 | 16.1 | 11 | 0-58 | 0.2409 |
| 2 (Fig. 4F) | *Δpbpimms2*::*pimms2*$^{mut}$ | 4 | 108 | 63 | 14 | 5 | 0-142 | |
| | *Δpbpimms2* | 4 | 127 | 71 | 8 | 3 | 0-120 | 0.0172 |
| | *Δpbpimms2*::*pimms2*$^{wt}$ | 4 | 97 | 75 | 27 | 10 | 0-199 | 0.0031 |
| | *wt* | 4 | 89 | 73 | 40 | 13 | 0-253 | 0.0007 |

The table reports results of genetic complementation experiments reported in Figures 4D and 4F, respectively. For each experiment series, infected *A. gambiae* midguts from independent biological replicates (2 and 4, respectively) were pooled for each group. The numbers of midguts in each replicated are shown in the fourth column. Midguts with zero parasites were also considered for calculation of the arithmetic mean and median of infection intensities. The prevalence shows the percentage of midguts with at least one oocyst. *P* values were calculated using the Mann-Whitney *U*-test and presented for comparisons between *Δpbpimms2*::*pimms2*$^{wt}$ and *Δpbpimms2 or wt* for the first series of infections and between *Δpbpimms2*::*pimms2*$^{mut}$ and *Δpbpimms2* or *Δpbpimms2*::*pimms2*$^{wt}$ or *wt* for the second series of infections. The *P* value of the comparison between *wt* and *Δpbpimms2*::*pimms2*$^{wt}$ is 0.3474.

**Table S4**. Primers for RT-PCR, generation of *transgenic* parasites and protein expression.

| **Primer name** | **Sequence (5′ to 3′)** | **Description** |
|---|---|---|
| *PbPIMMS2* RT-PCR F | GACAATCCAAGGCGATTCAT | |
| *PbPIMMS2* RT-PCR R | GCAATATCAGCGTCATCAGAA | |
| *P28* RT-PCR F | AATGCACAGGTACAGGAGAAACTAAAT | |
| *P28* RT-PCR R | CACACTCATAATGTTTTCCAGTCAATT | |
| *AMA1* RT-PCR F | TATGGGTCCAAGATATTGTAGTAATAA | |
| *AMA1* RT-PCR R | GAATTAGCTTTACCATAAATATCTGC | |
| *CHT1* RT-PCR F | GCCCGCCCAGATGTAATTATA | |
| *CHT1* RT-PCR R | TGCCAAATTCCTACACCATCG | |
| *PbPIMMS2* a (P1) | TT-GGGCCC-CGTTGTAAAATTGTCCAAACAAA | Disruption upstream target *ApaI* |
| *PbPIMMS2* b (P2) | CC-AAGCTT-ACACCCTTTGAAACATATTATCATCA | Disruption upstream target *HindIII* |
| *PbPIMMS2*c (P3) | T-GAATTC-ACTTTAAGATGGGGTTTAATTCAAAGA | Disruption downstream target *EcoRI* |
| *PbPIMMS2*d (P4) | TT-GGATCC-GTTTTCCGAACTAGGAAATTCATTAT | Disruption downstream target *BamHI* |
| *PbPIMMS2* INT F (P5) | AGCGTCTAGTAGTTTGAGCTAGCTA | 194 bp upstream of *PIMMS2* |
| *PbPIMMS2* WT R (P6) | GCAATATCAGCGTCATCAGAA | 802 bp downstream of *PIMMS2* |
| *TgDHFR* 5'UTR R (P7) | GATGTGTTATGTGATTAATTCATACAC | 200 bp into the 5'UTR of *TgDHFR-TS* |
| *PbPIMMS2* e (P8) | CC-AAGCTT-CGTTGTAAAATTGTCCAAACAAA | |
| *PbPIMMS2* f (P9) | GG-CCGCGG-TTATCTAGAATATTTCTTGGAATTA | |
| *PbPIMMS2* g (P10) | TT-CTCGAG-ACTTTAAGATGGGGTTTAATTCAAAGA | |
| *PbPIMMS2* h (P11) | TT-CCCGGG-GAAATTGTGTATTGTGTGAAAAATATA | |
| *SUBO* complement INT R (P12) | GAGGTTATATTAATTGGTAAACCATTG | |
| PbPIMMS2 D155A F (P13) | AACCTCAGTATGCTTAATAGCGACTGGATTGAATATTAAGGAT | |
| PbPIMMS2 H222A F (P14) | GAGAATCATAATGGGGCTGGAAAATATGAAGAT | |
| PbPIMMS2 S414A F (P15) | TATTATGAAATTAAATACAGTGCTGCATTTTTATAAATATAA | |
| PbPIMMS2$^{opt}$CD F (P16) | GACAAGCTTGCGGCCGCACCCATCAACATCACCTCCGTGTG | |
| PbPIMMS2$^{opt}$CD R (P17) | TGCTCGAGTGCGGCCGCGATACCGCGGTTCAGCAGGATGTG | |

Where appropriate, target restriction sites are shown as underlined italics and restriction site overhangs are also shown. The appropriate restriction enzyme is presented in the description column. F, forward; R, reverse. All primers are listed in a 5′ to 3′ direction.
